# Supplementary material for: Observing growth and interfacial dynamics of nanocrystalline ice in thin amorphous ice films
Source: Nat Commun. 2024 Jan 30;15:908. doi: 10.1038/s41467-024-45234-x (PMC10827800; doi:10.1038/s41467-024-45234-x)
Supplement: Supplementary file 1 — Supplementary Info [file 41467_2024_45234_MOESM1_ESM.pdf]

Supplementary information for

Observing growth and interfacial dynamics of nanocrystalline ice in thin amorphous ice films

Minyoung Lee[1,2]†, Sang Yup Lee[3,4,5]†, Min-Ho Kang[6,7]†, Tae Kyung Won[3,5], Sungsu Kang[1,2], Joodeok Kim[1,2], Jungwon Park[1,2,8,9]*, and Dong June Ahn[3,4,5]*

[1]School of Chemical and Biological Engineering, and Institute of Chemical Processes, Seoul National University; Seoul, 08826, Republic of Korea.

[2]Center for Nanoparticle Research, Institute of Basic Science (IBS); Seoul, 08826, Republic of Korea.

[3]Department of Chemical and Biological Engineering, Korea University; Seoul, 02841, Republic of Korea.

[4]KU-KIST Graduate school of Converging Science and Technology, Korea University; Seoul, 02841, Republic of Korea.

[5]The w:i Interface Augmentation Center, Korea University; Seoul, 02841, Republic of Korea.

[6]Department of Biomedical-Chemical Engineering, The Catholic University of Korea; Bucheon-si, 14662, Republic of Korea.

[7]Department of Biotechnology, The Catholic University of Korea; Bucheon-si, 14662, Republic of Korea.

[8]Institute of Engineering Research, College of Engineering, Seoul National University; Seoul, 08826, Republic of Korea.

[9]Advanced Institutes of Convergence Technology, Seoul National University; Suwon-si, 16229, Republic of Korea.

*Corresponding author. Email: ahn@korea.ac.kr and jungwonpark@snu.ac.kr

†These authors contributed equally to this work.

Supplementary Text

1. Electron beam effects on amorphous ice film crystallization

Control experiments were performed to compare the image results of heat-induced and beam-induced crystallization processes that are manifested differently in our experimental conditions. Heat-induced ice crystallization shown in Supplementary Fig. 3a produces ice domains that are mainly higher in contrast than the amorphous ice, while the larger domains have white regions surrounding them, which are the bright contrast interfaces we have mentioned in our main text. We note that in heat-induced ice crystallization, we have imaged different locations of ice films which were exposed to different annealing times at 143 K to minimize the electron beam exposure. At 93 K, continuous irradiation of the electron beam on amorphous ice results in beam-induced crystallization as shown in Supplementary Fig. 3b. At a threshold dose of 137 e^{-} Å^{-2}, dark and bright spots, marked with blue and yellow arrows respectively, form on the ice film. These spots eventually grow into domains that exhibit either black or white contrast compared to the amorphous portion of the film. The contrast differs according to the alignment of the domains with respect to the electron beam, which depends on the diffraction conditions (Supplementary Fig. 4) that change with stage tilt. These domains do not exhibit the bright contrast regions we have observed with heating-induced crystallization. Previous studies on heat-induced crystallization of amorphous ice have reported that the increased mobility of the molecules throughout the entire ice film beyond 136 K lowers the thermodynamic driving force for nucleation and growth, resulting in crystallization[2,3]. Electron beam-induced crystallization, on the other hand, is caused by (1) enhanced mobility of water molecules due to added beam energy and (2) local heating effects[4], which leads water molecules to crystallize at local, affected regions of the amorphous ice.

To understand the combined effects of heating and beam-induced crystallization, electron beam irradiation experiments were performed at 143 K, as shown in Supplementary Fig. 3c. The first

image shows the presence of domains, some with bright contrast regions that are characteristic of heat-induced crystallization. At a threshold dose of 22.2 e^{-} $Å^{-2}$, dark spots due to beam-induced crystallization start to form amidst amorphous ice. We could observe much faster and frequent depletion of amorphous ice due to nucleation of beam-induced crystals at 143 K, because water molecules exhibit higher mobility at this temperature[4]. Using these results on the threshold dose, we have excluded TEM data obtained at 143 K that has been exposed to more than 22.2 e^{-} $Å^{-2}$ of electron dose in the main text to ensure reliability of the data.

2. Elemental analysis of amorphous ice films and ice nanocrystals

To confirm that the high-contrast features present at 143 K are indeed crystalline ice and not from impurities, we performed identical-location TEM. This process involves taking an image of a hole with amorphous ice film at 93 K, and then heating the sample to 143 K with the beam blanked, before taking another image of the same hole at 143 K. A representative image of the amorphous ice hole at 93 K exhibits uniform contrast without dark contrast features that may otherwise indicate the presence of impurities such as metal nanoparticles or ice deposits (Supplementary Fig. 5a). The domains formed at 143 K are formed solely from amorphous ice. We have also observed some ice films containing very dark ice deposits at 93 K before the heating process. A representative example is shown in Supplementary Fig. 5b. We note that images having these sorts of dark deposits were excluded from quantification. We have also utilized EDS elemental mapping of an amorphous ice region shown in Supplementary Fig. 5c to confirm that no other elements other than C from the carbon film, Cu, Fe, Co from the holder, and Au from the grid were present, and therefore, there were no potential sources of contaminants such as NaCl (Supplementary Fig. 5d).

EELS spectroscopy also allows for the characterization of phases and mapping of elements. Supplementary Fig 5e is an ADF-STEM image for acquisition of low loss EELS spectra of amorphous ice with crystalline domains grown. Low loss EELS spectra were obtained in a

region with an ice domain (green box and spectra). The domain region exhibits a shoulder peak at 9 eV in the core loss region, attributed to the electronic interband transition of ice[1]. Core loss EELS mapping was also performed on an amorphous ice hole to rule out carbon-based contaminants. Three representative spectra obtained at different positions within a hole are shown in Supplementary Fig 5g, in which we confirm that there are no C K edge peaks (284 eV) and only O K edge peaks (540 eV) from H_2O molecules are present.

3. TEM simulations for interpretation of HRTEM images

Extensive TEM image simulations on cubic, hexagonal, and stacking defect models were performed, in which the thickness and defocus values of the sample had been varied to find conditions that match the TEM images. Supplementary Fig. 12a and b shows the raw and low-passed filtered HRTEM images, respectively, used for comparing TEM simulation data. Areas 1, 2 and 3 marked in Supplementary Fig. 12b were specifically targeted because these are areas that were indexed as cubic, hexagonal, and stacking defect sequences, respectively in Fig. 2e and f. Representative images of TEM simulation results for the sequences with stacking defects, modelled by using Area 3 marked in Supplementary Fig. 12b, are shown in Supplementary Fig. 12c. As a result, we found that the stacking faults of ice crystals shown in Area 3 are well-manifested in the simulated TEM images with sample thickness of 100 nm, defocus values of 80 to 90 nm, and tilt of 1.5 mrad, Cs = 1.5 mm and Cc = 1.1 mm. Using these simulation conditions, we have performed TEM simulations on Areas 1, 2 and 3, which contains the regions in which we marked the atomic positions for I_c, I_h, and I_{c+h} sequences, respectively. We found that the experimental data matches well with our simulated TEM data (Supplementary Figs. 12d and e), and with our imaging conditions, the atom positions are bright in contrast as the white spots correspond to the atomic positions in the exit wave images (Supplementary Fig. 12f). Through these newly performed simulation results that better match our experimental conditions, we can conclude that the white spots in our high resolution TEM images can be

compared to the atomic positions in our atomic models (Supplementary Fig. 12g), showing the presence of cubic, hexagonal sequences, as well as stacking defects in heterocrystalline ice.

4. HRTEM analysis for the interfacial area

The HRTEM image of the interfacial area of a fast-growing ice domain was imaged as shown in Supplementary Fig. 17a, and a false-colored, low-pass, gaussian filtered image of the close-up of the boxed interfacial area is shown in Supplementary Fig. 17b. The cubic sequences and the stacking faults are visible in the HRTEM image at the crystalline region. For crystalline regions of the ice, the well-aligned atomic columns enable visibility of the atomic structures. The atomic positions are not clearly defined at the interfacial region, which is thick and amorphous in nature. According to our TEM simulation results with water molecules having a density of 1.0 g/cm^3 (Supplementary Fig. 17c), the resulting simulated images obtained with a perfect detector free of noise, and with a CCD both do not allow us to specify the exact positions of the oxygen atoms (Supplementary Fig. 17d).

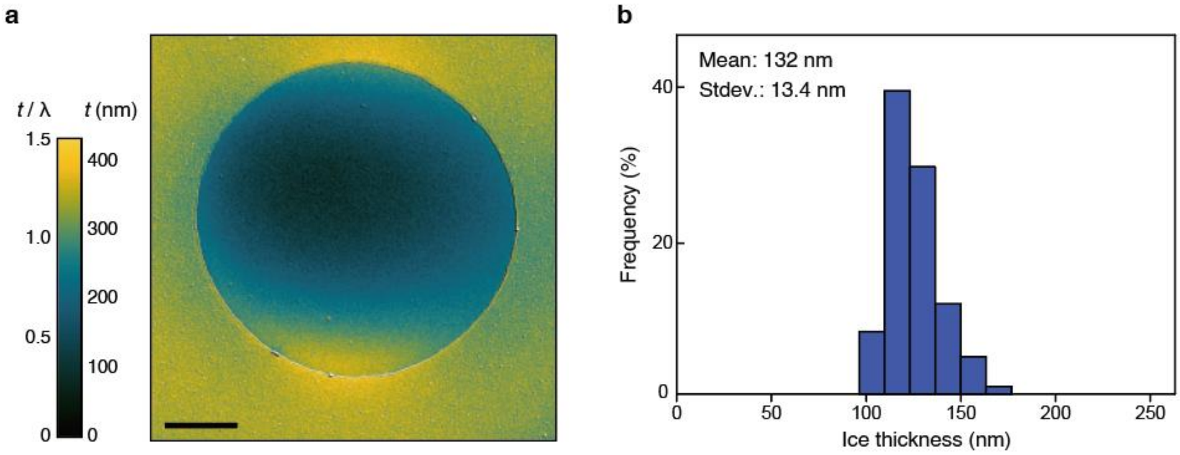

Supplementary Fig. 1: EFTEM analysis for amorphous ice thickness measurement.
a EFTEM image of a carbon film hole with a free-standing amorphous ice film in the region inside the hole (Scale bar = 200 nm). Each pixel is color-mapped based on t/λ values. The thickness t is calculated based on the inelastic mean free path value of ice 287 nm at 200 keV. **b** Histogram of ice thickness values obtained for each pixel located inside holes, taken from multiple holes.

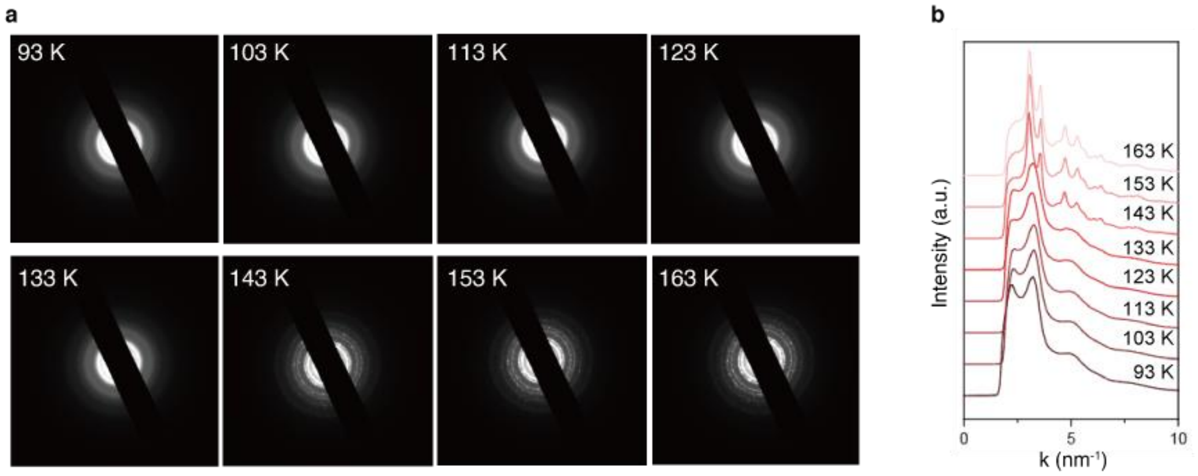

Supplementary Fig. 2: Characterization of ice films before and after ice nanocrystal growth.

SAED was performed at a fixed location in the amorphous ice film while increasing the temperature. Note that the beam was blanked in between acquisitions. **a** SAED patterns of the same region of ice at different temperatures. **b** Radial averages of SAED patterns. Crystalline peaks emerge starting from 143 K, which was the temperature chosen to perform the ice annealing experiments on.

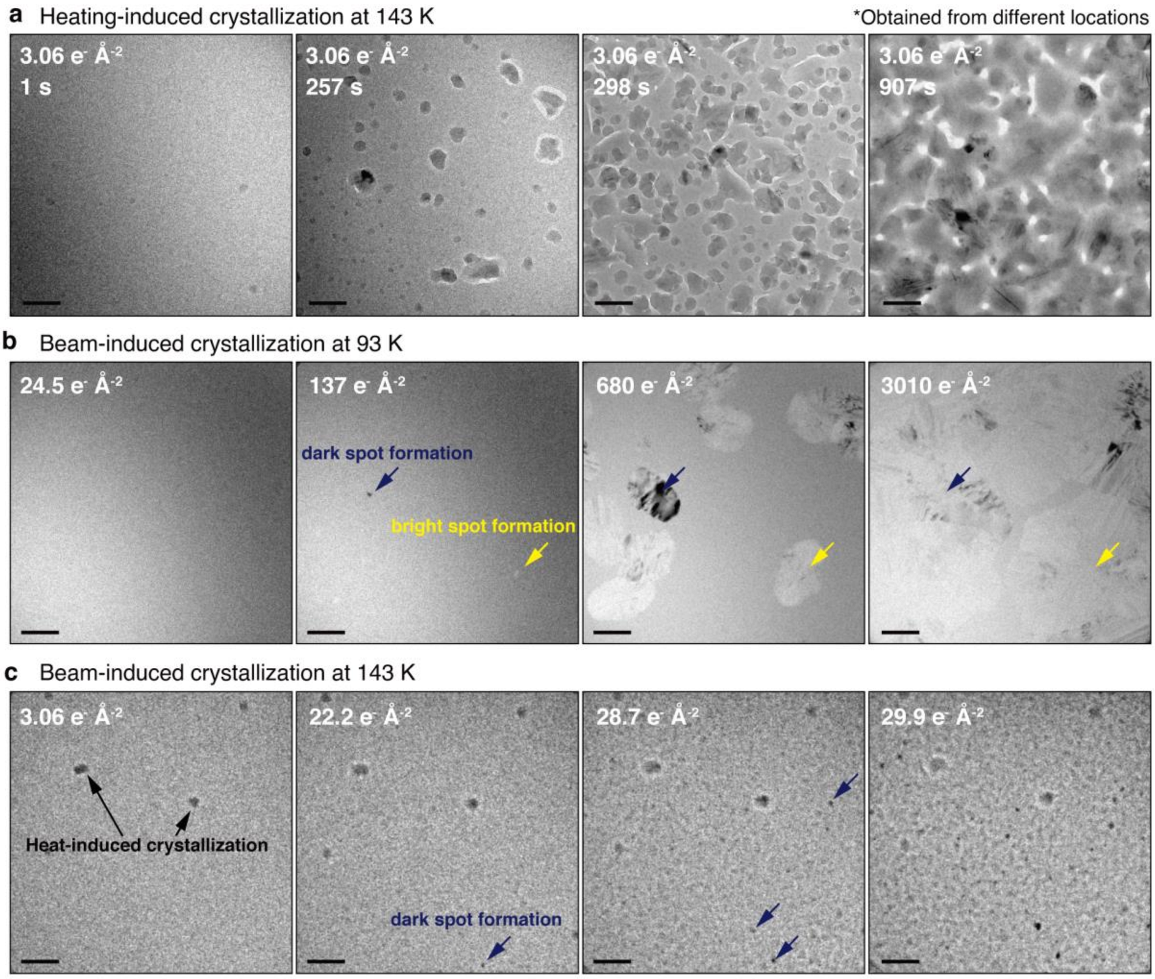

Supplementary Fig. 3: Comparison between heating-induced and beam-induced crystallization.
a Heating-induced crystallization at 143 K, in which each image was obtained at different locations of the film to minimize electron beam exposure. **b** Beam-induced crystallization at 93 K, caused by continuous irradiation of the electron beam on the amorphous ice film. **c** Beam-induced crystallization at 143 K, examining the combined effects of heating amorphous ice to 143 K and the electron beam. Scale bars = 100 nm.

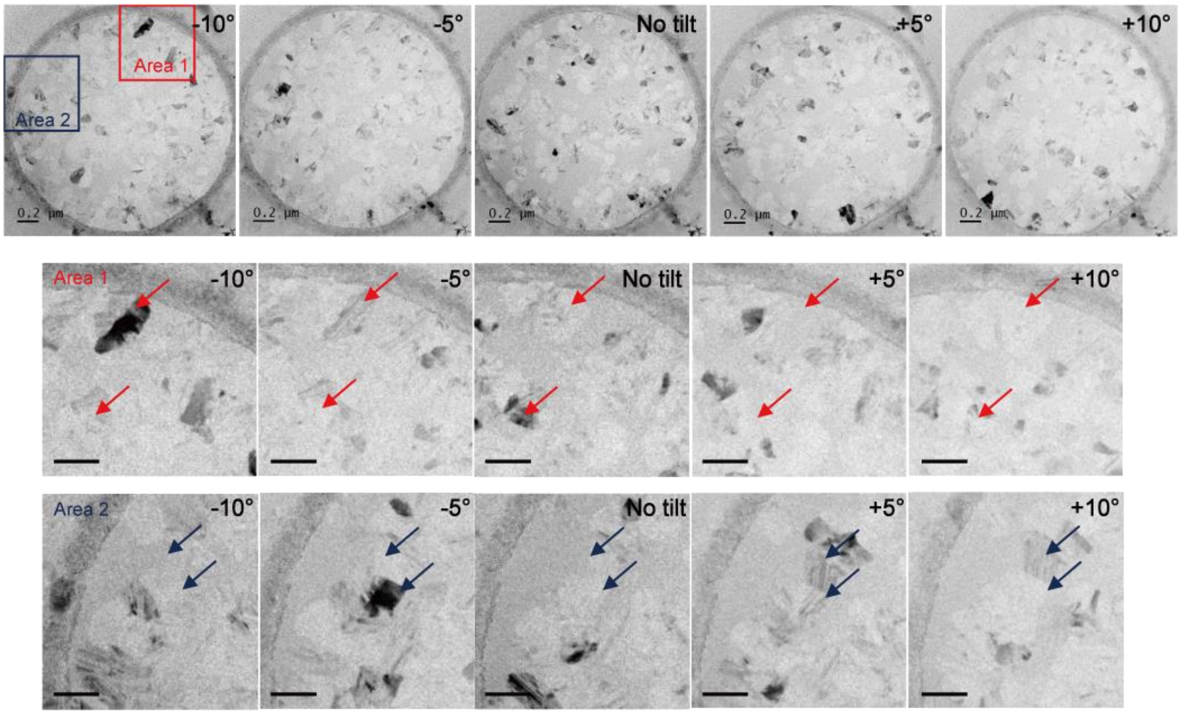

Supplementary Fig. 4: Electron beam-induced crystallization of amorphous ice observed with specimen tilting.
Electron beam-induced crystallization was performed on an amorphous ice film at 93 K, and the image was tilted to change the Bragg planes of the crystals relative to the incident beam. Arrows mark the particles that change in diffraction contrast with incident beam angle. Scale bars = 200 nm.

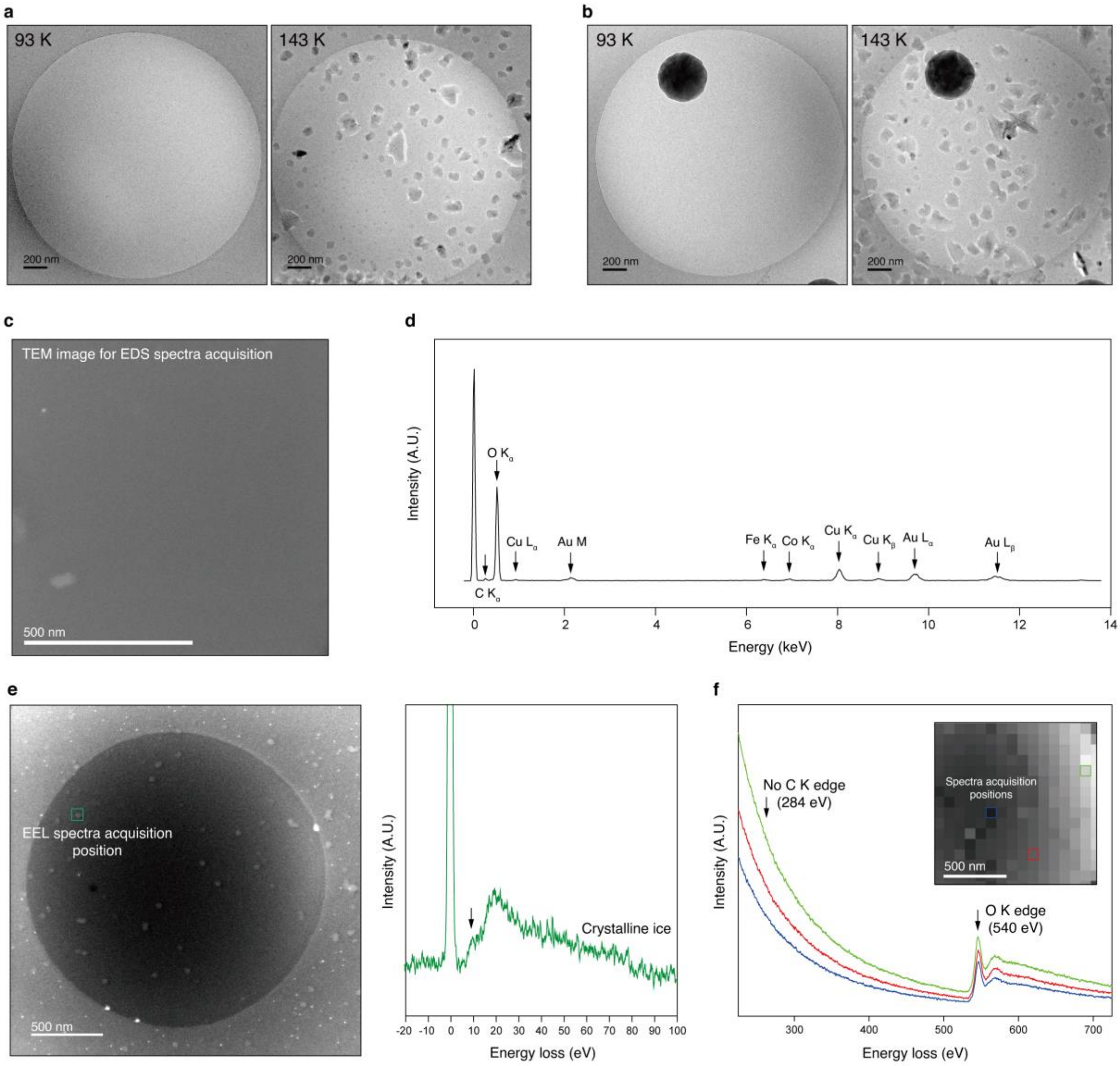

Supplementary Fig. 5: Elemental analysis of ice nanoparticles.
a,**b** Identical-location TEM results at 93 K (left) and 143 K (right). **a** shows a representative region used for quantitative analysis, and **b** shows a region with an adsorbed ice vapor contaminant, which was excluded during analysis. **c** HAADF-STEM image and **d** EDS spectrum obtained from elemental analysis of the region in **c**, showing that no other elements except for O from ice, C from the carbon film, Au from the grid, Cu, Fe, and Co from the holder. **e** ADF-STEM image of ice particles grown in amorphous ice (left) and low-loss spectra (right) obtained in the green box in **c** for a crystalline ice domain. The crystalline ice domain exhibits a shoulder peak at 9 eV, indicative of ice. **f** Core loss spectra at the region of the C K edge and O K edge, showing that no carbon peak is present and that there are no carbon contaminants. The inset shows the spectrum map.

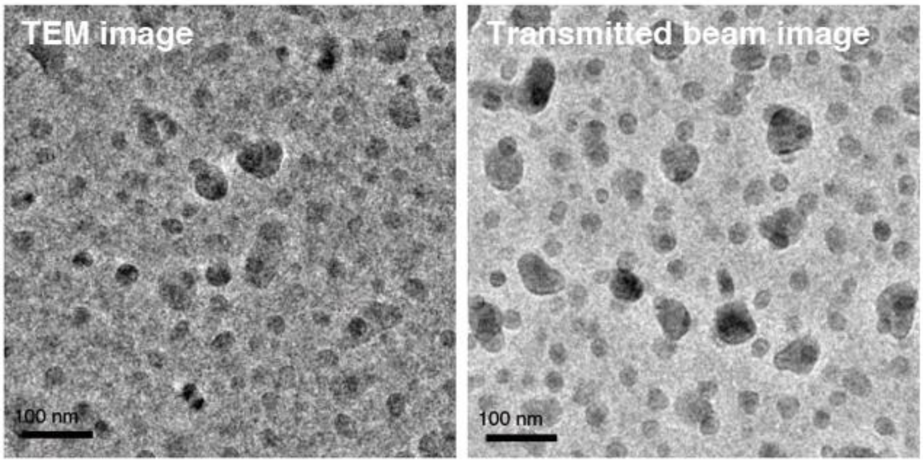

Supplementary Fig. 6: Comparison between conventional TEM and BFTEM
Comparison between conventional TEM imaging and transmitted-beam imaging (bright field TEM, or BFTEM) shows that the latter exhibits higher contrast, aiding delineation of particles.

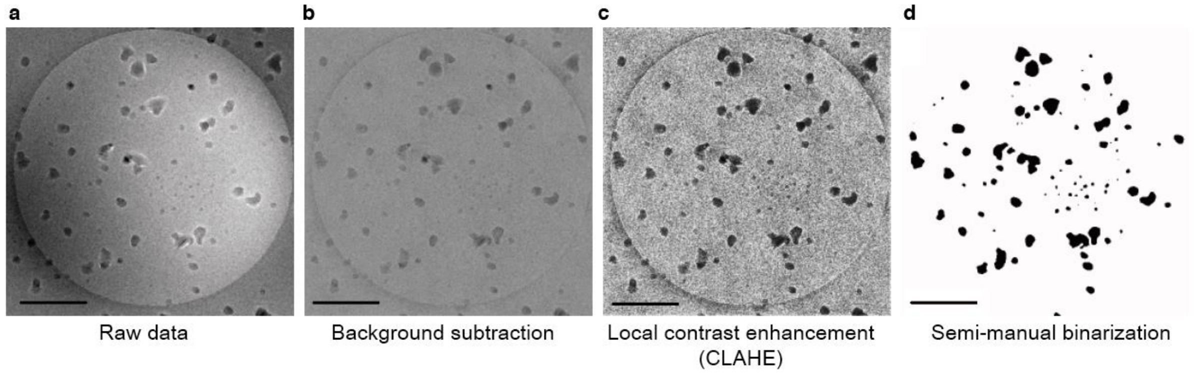

Supplementary Fig. 7: Contrast enhancement and binarization of ice nanoparticles.
a Raw data from cryo-EM imaging. **b** Image after background subtraction, **c** applying local contrast enhancement using CLAHE algorithm, and **d** binarized particles using thresholding and manual selection. Scale bars = 500 nm.

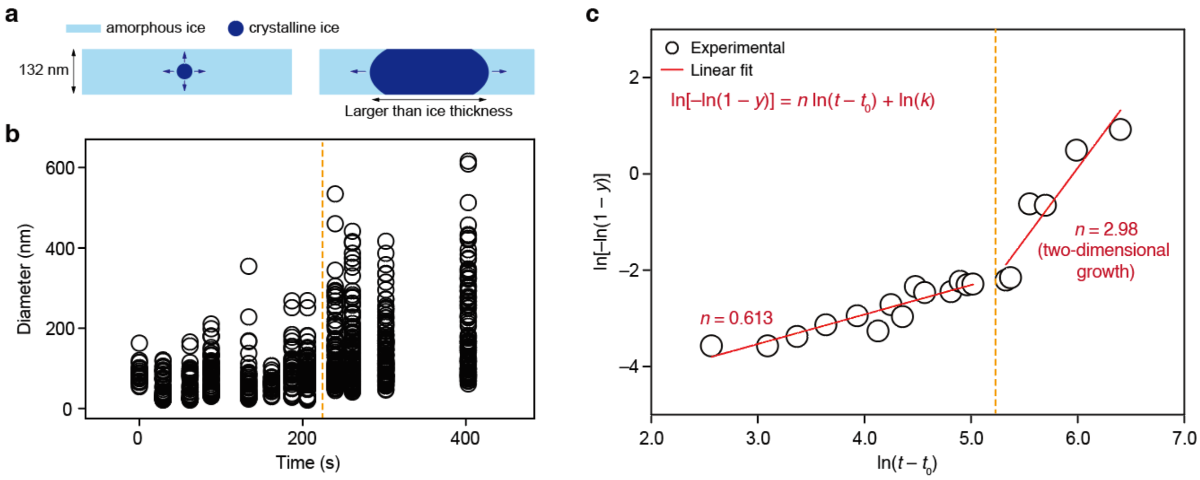

Supplementary Fig. 8: Analysis for growth modes of ice domains according to different sizes.

a Schematic of the proposed growth mechanisms of ice nanocrystals of different sizes. **b** Distributions of Feret diameter of ice nanocrystals according to annealing time in between 0 s and 402 s, where we identify the point in time in which particle sizes undergo a sudden increase in diameter, which occurs after 215 s of annealing. **c** Log-log graph of the Avrami equation, which was calculated with the crystallized fraction and the annealing time. The graph is fit with two separate regimes of growth delineated by the yellow dotted line, fitted with n = 0.613 and n = 2.98 (red lines).

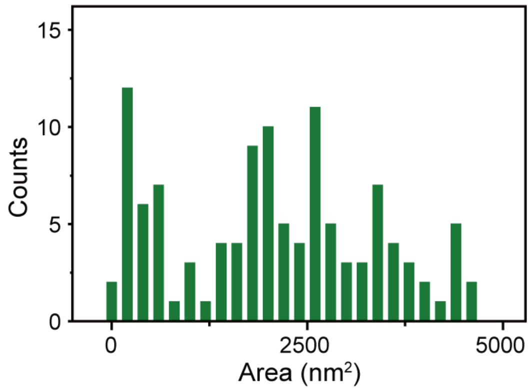

Supplementary Fig. 9: Size distribution of small, finitely growing ice domains. The size distribution histogram was obtained from the image in Fig. 1h, characterized as ice $I_{c.}$

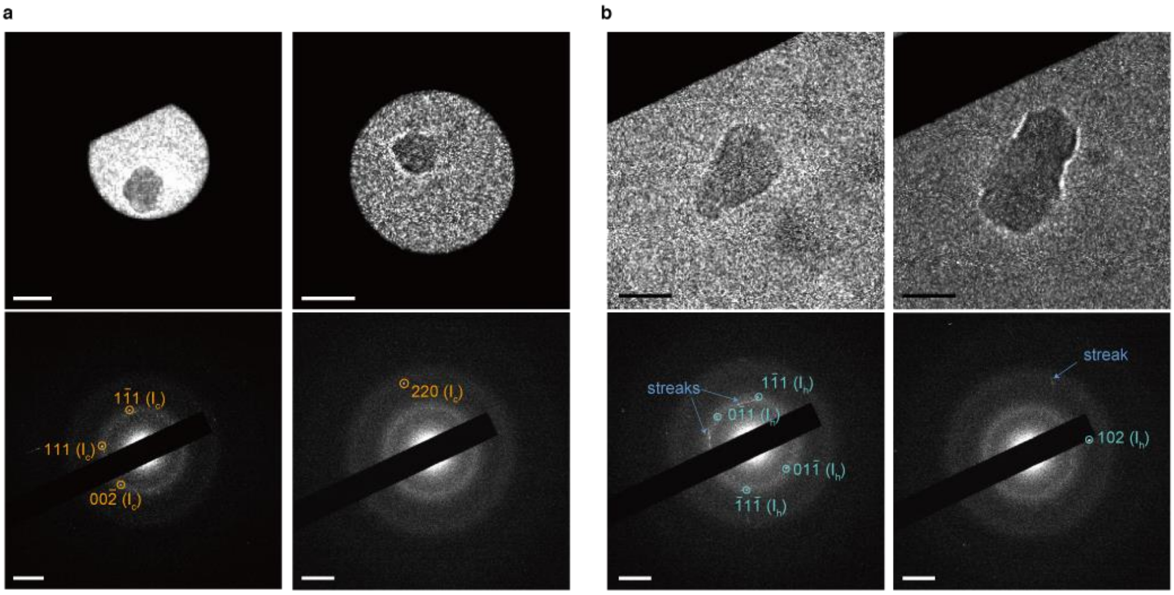

Supplementary Fig. 10: Diffraction patterns of individual I_c and I_{c+h} nanocrystals. **a**,**b**, Representative TEM images and the corresponding SAED patterns of **a** ice I_c and **b** ice I_{c+h} nanocrystals. Scale bars for images = 50 nm, scale bars for SAED patterns = 2 nm^{-1}.

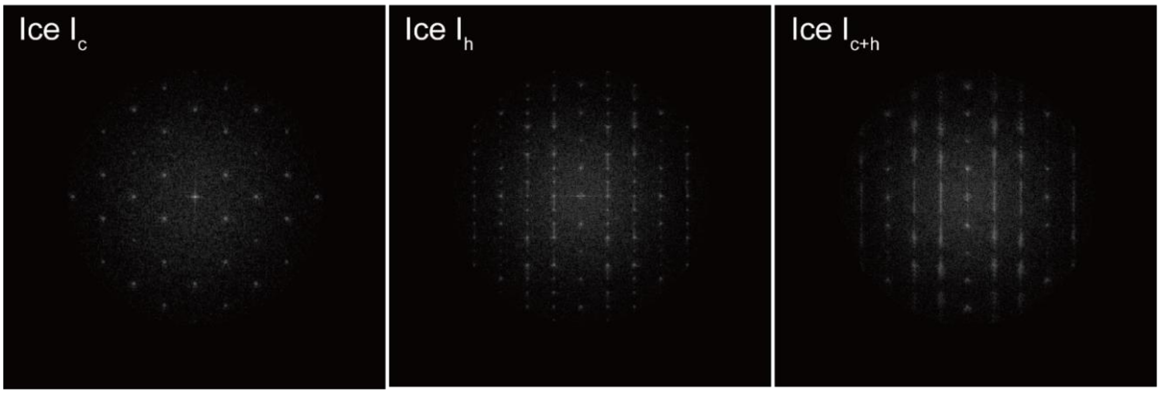

Supplementary Fig. 11: Electron diffraction simulations of I_c, I_h, and I_{c+h} models. Results for the simulations of electron diffraction patterns for ice I_c (left), I_h (center), and I_{c+h} (right).

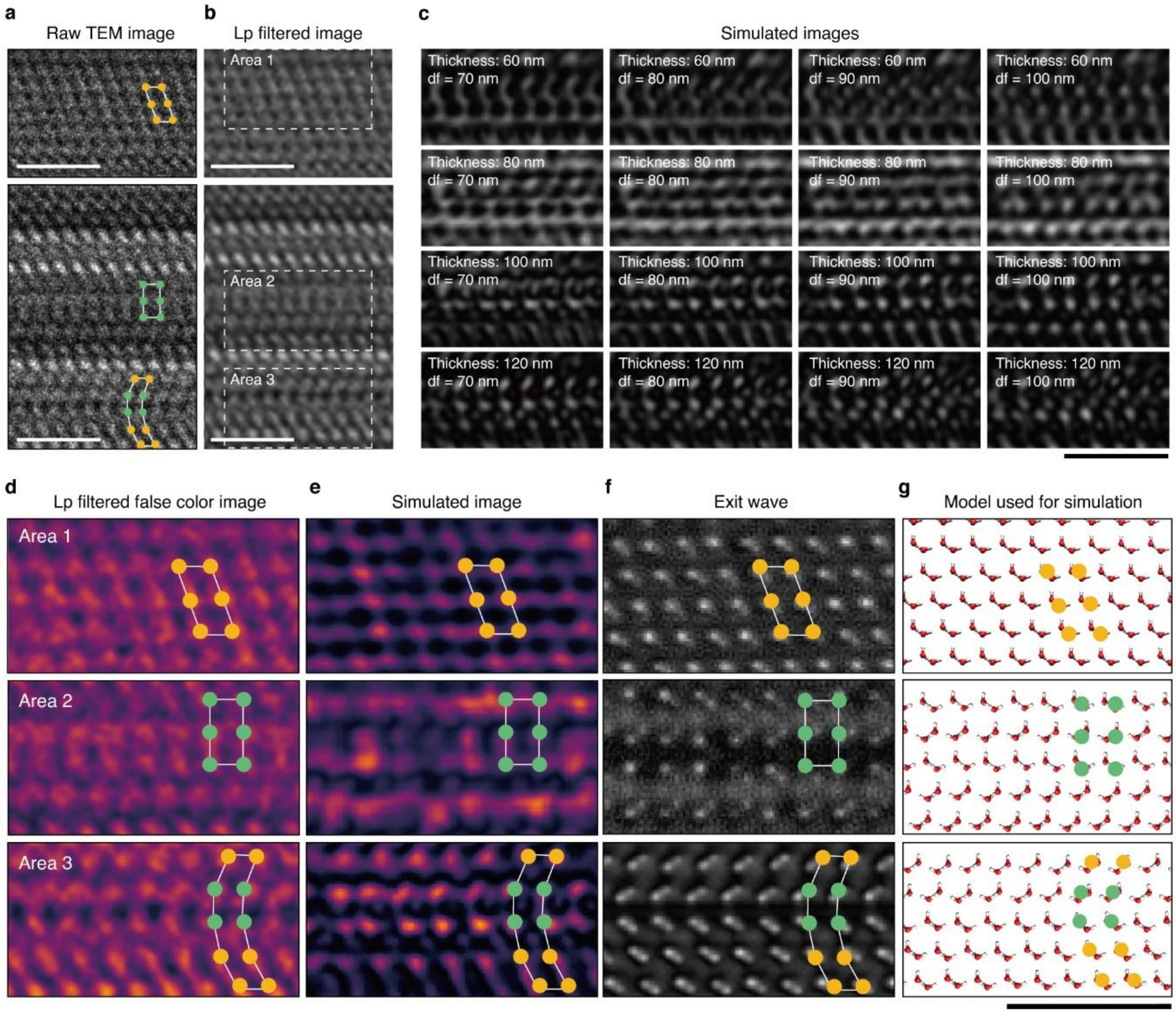

Supplementary Fig. 12: Experimental and simulated HRTEM images determining the structural features in heterocrystalline ice.

a The raw, original high-resolution TEM image of a grown heterocrystalline ice I_{c+h} domain at the [110] zone axis, with cubic (yellow circles), hexagonal (green circles), and stacking defect sequences (cubic and hexagonal) marked, as presented in the original manuscript. **b** Low-pass filtered high-resolution image to reduce high-frequency noise. Areas marked with the white dotted square have been compared with TEM simulations to verify the accuracy of the atomic position labels in **a**. **c** Representative TEM simulated images for different ice slab thickness and defocus values, modelled with the image shown in Area 3. Thickness of 100 nm and defocus values ranging from 80 to 90 nm show the best match to the experimental TEM image of Area 3. TEM simulations were performed with the multislice algorithm, with Cs = 1.5 mm and 1.5 mrad tilt. **d**,**e**,**f**,**g** Results of TEM simulations. Cubic (yellow circles), hexagonal (green circles), and stacking defect sequences (cubic and hexagonal) are labelled. **d** False-colored images of Areas 1, 2 and 3 labelled in **b**. **e** TEM simulated images, performed with thickness of 100 nm and defocus ranging from 80 to 85 that best match the images in **d**. **f** Exit wave images indicating the positions of oxygen atoms in white. **g** Models used for simulation. Scale bars = 2 nm.

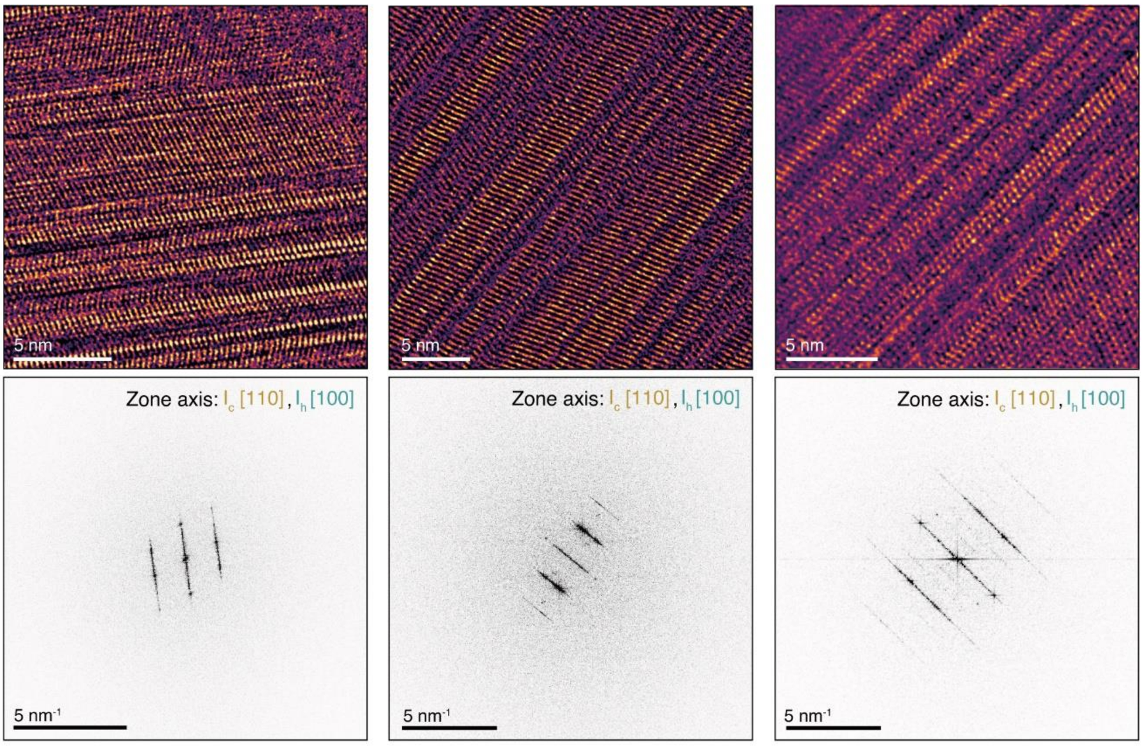

Supplementary Fig. 13: High resolution TEM images of heterocrystalline ice.
Top) False-colored high resolution images of fast-growing heterocrystalline ice, showing lines of different contrast indicating the presence of heterocrystallinity and defects. Bottom) Corresponding FFT obtained from TEM images. The presence of streaks in the FFT indicate the presence of stacking defects.

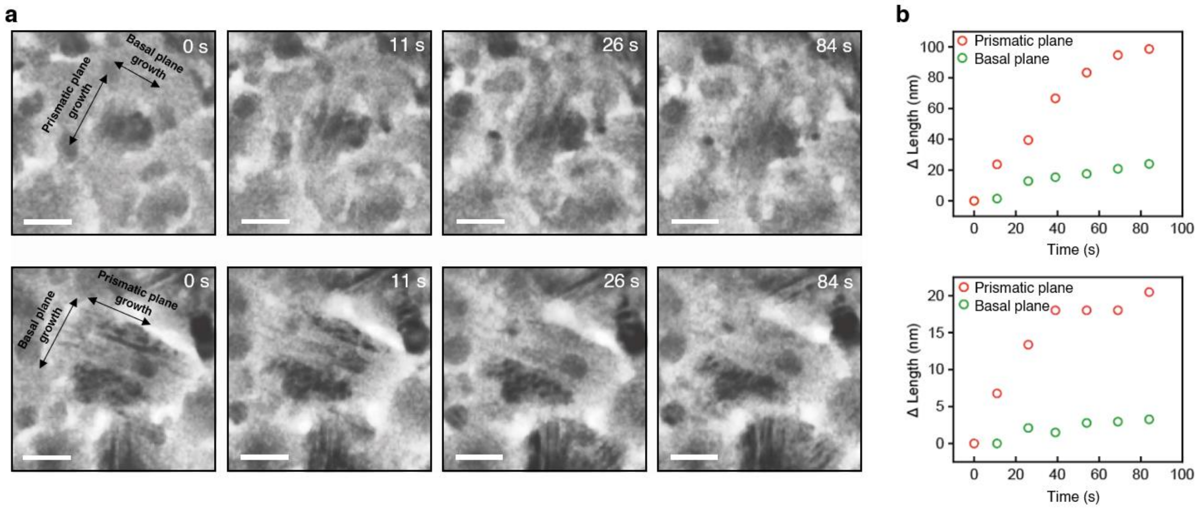

Supplementary Fig. 14: Anisotropic growth of ice I_{c+h} nanocrystals.
a *In-situ* BFTEM time-series images of ice I_{c+h} with streaks. **b** The displacement of corresponding domain edges along the prism and basal plane directions, determined by the direction of the streaks. Scale bars = 100 nm.

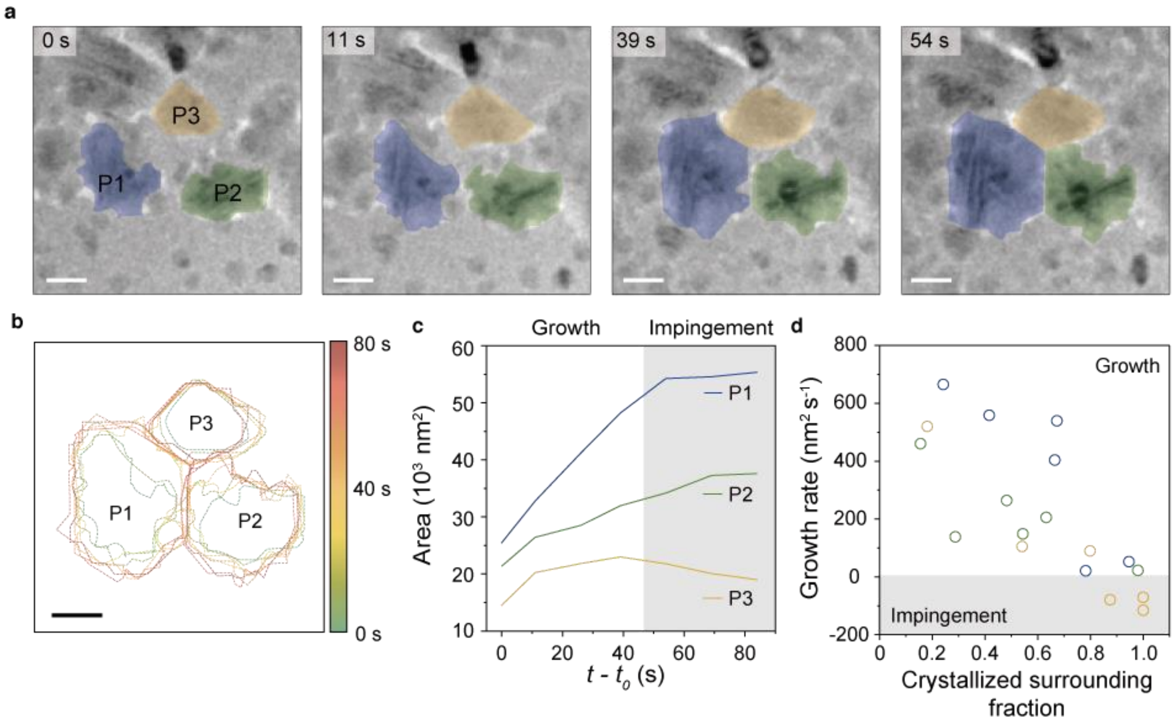

Supplementary Fig. 15: Impingement of I_{c+h} domain boundaries.
a *In-situ* BFTEM time-series images of ice I_{c+h} particles labelled P1, P2 and P3 undergoing growth and impingement of boundaries at later stages of growth. **b** Contours of P1, P2, and P3 over time. **c** Areas of P1, P2, and P3 plotted over time. **d** Growth rates of P1, P2, and P3 as a function of the extent of the surrounding that has crystallized. The results show that boundary impingement affects the growth rate of ice I_{c+h} domains. Scale bars = 100 nm.

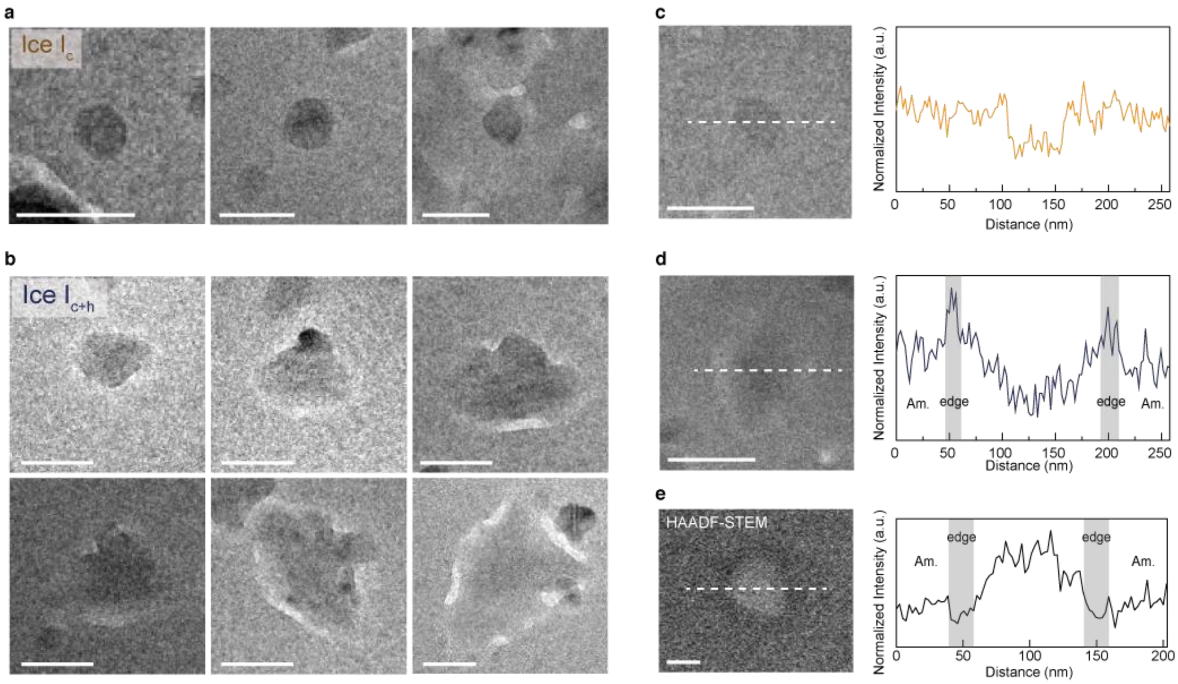

Supplementary Fig. 16: Bright-contrast regions near crystalline-amorphous interfaces of continuously growing ice I_{c+h} domains.
a Representative BFTEM images of ice I_c and **b** pixel intensity line profile for an ice I_c domain, exhibiting no bright-contrast regions at the interface. **c** Representative BFTEM images of ice I_{c+h} and **d** pixel intensity line profile for an ice I_{c+h} domain, exhibiting bright-contrast regions at the interface. **e** HAADF image and pixel intensity line profile for an ice I_{c+h} domain, exhibiting mass thickness contrast at the edges of the domain. Scale bars = 50 nm.

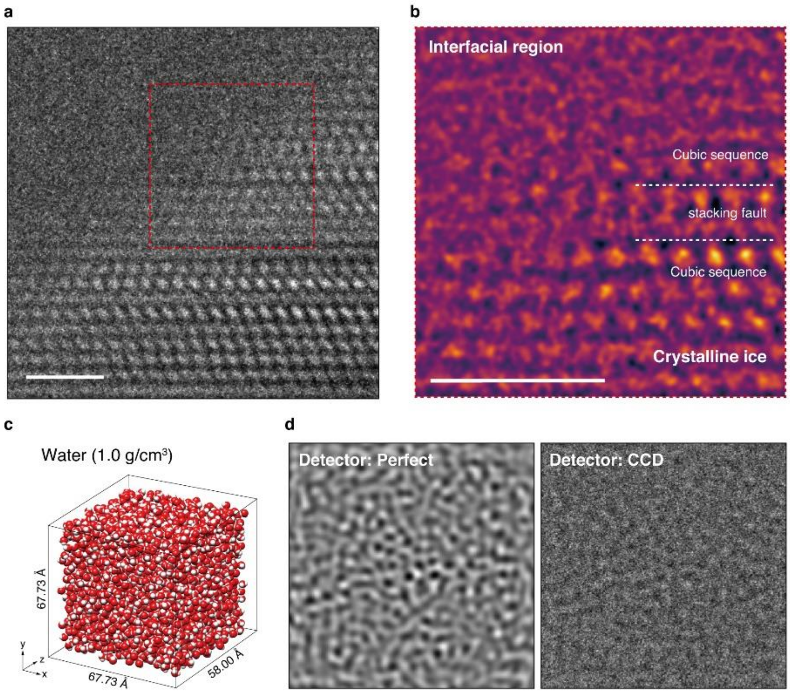

Supplementary Fig. 17: HRTEM image of the interfacial area.
a HRTEM image of an interfacial area of a crystalline ice particle. **b** false color close-up image of the region marked as the red, dotted box in **a** (Scale bars = 2 nm). The image has been low-pass filtered, and then a gaussian filter has been implemented. **c** Water model with density 1.0 g/cm^3 obtained with MD simulations. **d** Simulated TEM images of water molecules with 58 Å thickness. Even with the perfect detector, which is free of noise, the position of water molecules are not defined.

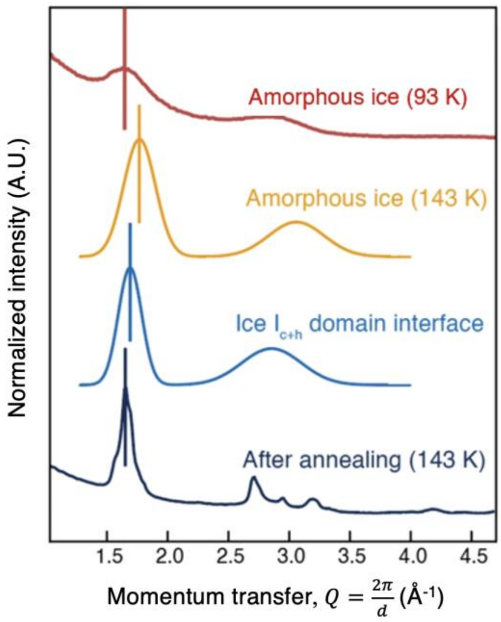

Supplementary Fig. 18: Radial averages of electron diffraction patterns.
Radial averages of SAED patterns of amorphous ice at 93 K (red), amorphous ice at 143 K (yellow), the ice I_{c+h} domain interface (light blue) and crystalline ice after annealing (dark blue). Peak positions of the first peak are marked with a horizontal line for comparison.

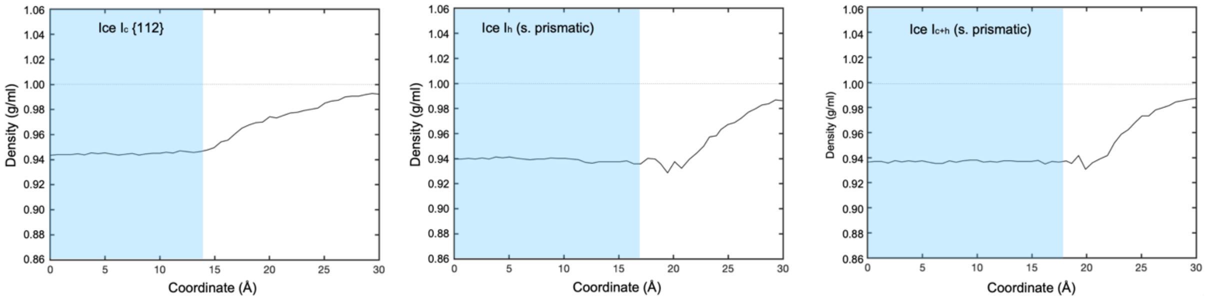

Supplementary Fig. 19: Local coarse-grained density profile at water-ice interface. Bulk density profile of water molecules at interface around the ice I_c {112} (left panel), the ice I_h (s. prismatic) (center panel), and the ice I_{c+h} (s. prismatic) (right panel). The area shaded in blue represents the ice region. A 5-angstrom slab was used.

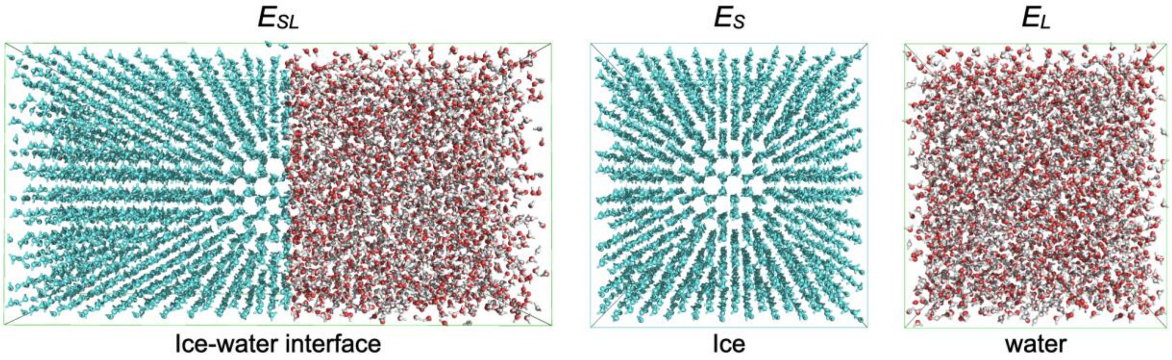

$$\gamma_{SL} = \frac{E_{SL} - E_L - E_S}{2A} - \frac{T\Delta S}{2A}$$

Supplementary Fig. 20: Calculation of ice-water interfacial tension.
Illustration of the method to calculate the solid-liquid interfacial tension (γ_{SL}) using three boxes with an ice-water interface (E_{SL}), ice (E_S), and water box (E_L). The interfacial tension is calculated from the potential energy (E) of the three boxes, the entropy contribution (S), the temperature (T), and the surface area (A).

Ice planes	Solid-liquid interfacial energy @233 K (mJ/m^2)		
	Ice I_c	Ice I_h	Ice I_{c+h}
**Basal plane**	22.7 ± 0.7	22.3 ± 0.9	22.4 ± 0.8
**Secondary prism plane**	22.1 ± 0.7	23.6 ± 0.6	25.4 ± 0.7

Supplementary Table 1: Estimation of solid-liquid interfacial energies. Interfacial energies for basal and secondary prism planes were estimated. Ice I_{c+h} was produced as a structure with half cubic and half hexagonal sequences.

Supplementary References

1. Huang, X. *et al.* Tracking cubic ice at molecular resolution. *Nature* **617**, 86–91 (2023).

2. Löfgren, P., Ahlström, P., Lausma, J., Kasemo, B. & Chakarov, D. Crystallization kinetics of thin amorphous water films on surfaces. *Langmuir* **19**, 265–274 (2003).

3. Smith, R. S., Huang, C., Wong, E. K. L. & Kay, B. D. Desorption and crystallization kinetics in nanoscale thin films of amorphous water ice. *Surf. Sci. Lett.* **367**, L13–L18 (1996).

4. Xu, H., Ångström, J., Eklund, T. & Amann-Winkel, K. Electron Beam-Induced Transformation in High-Density Amorphous Ices. *J. Phys. Chem. B* **124**, 9283–9288 (2020).
