# Supplementary material for: Observing growth and interfacial dynamics of nanocrystalline ice in thin amorphous ice films
Source: Nat Commun. 2024 Jan 30;15:908. doi: 10.1038/s41467-024-45234-x (PMC10827800; doi:10.1038/s41467-024-45234-x)
Supplement: Supplementary file 3 — Description of Additional Supplementary Files [file 41467_2024_45234_MOESM3_ESM.pdf]

## Description of Additional Supplementary Files:

**Supplementary Movie 1:** Growth of ice nanocrystal domains in amorphous ice Scale bars = 50 nm.
